# Experimental-based statistical models for the tensile characterization of synthetic fiber ropes: a machine learning approach

**DOI:** 10.1038/s41598-023-44816-x

**Published:** 2023-10-18

**Authors:** Yahia Halabi, Hu Xu, Zhixiang Yu, Wael Alhaddad, Isabelle Dreier

**Affiliations:** 1 School of Civil Engineering, Southwest Jiaotong University, Chengdu, 610031 Sichuan China; 2 Department of Structural Engineering, Tongji University, Shanghai, 200092 China; 3 Institute for Lung Health (ILH), University of Giessen, Giessen, 35392 Hessen Germany

**Keywords:** Engineering, Materials science

## Abstract

This study investigated the tensile behavior of some prevalent synthetic fiber ropes made of polyester, polypropylene, and nylon polymeric fibers. The aim was to generate well-documented experimental statistics and develop simplified stress–strain constitutive laws that can describe the ropes' tensile response. The methodology involved analyzing the thermal history of the fibers using the DSC technique, tensile testing of fibers and yarn components of the rope, and conducting 196 rope tensile tests with optimum testing conditions. Based on the test results, an experimental database of the ropes' tensile characteristics was established, containing different parameters of material properties, rope construction, fiber processing, fiber tensile properties, and rope tensile responses. Subsequently, ANN models were developed and optimized using MATLAB based on the generated dataset's inputs and outputs to predict the studied ropes' tri-linear stress–strain profiles. The results showed that the ANN models accurately predicted the stress–strain properties of ropes represented by the tri-linear approximation with an error of about 5% for the failure strength and strain. The study provides insight into the *process-structure–property* relationship of synthetic fiber ropes and contributes to minimizing the cost and effort in designing and predicting their tensile properties while contributing to the practical industry.

Synthetic fibers are ubiquitous and have flexible characteristics, allowing them to be converted into various structures^1^. In particular, fibers are primordial constituents for making ropes by extruding and orienting the different polymers to provide the textile yarns, which are the starting point in rope production^2^. As polymeric substances, the mechanical properties of fibers and filaments are complicated, where the stressed fiber represents a sophisticated viscoelastic system^3^. Notwithstanding this complexity, the predominant mechanical properties of fibers in the direction of its axis are; the tensile modulus, tensile strength (tenacity), and elongation at break^4^. Most simply, the tensile stress–strain diagrams can determine these properties. However, rope tensile strength is the most apparent peculiarity for several applications. For instance, rope usage in the mooring demands^5–7^, netting structures^2^, energy absorbers in the personal fall protection vertical lifelines^8^, replacing the metal ropes in the tendon-driven robots^9^, strengthening the concrete columns through confinement^10–12^, and improving the seismic performance of the masonry walls^13^. Considering these different applications, it was incumbent upon the researchers.

to implement experimental, analytical, and numerical approaches to study the fiber ropes' mechanical properties. For instance^14^, established a consistent experimental database for twisted polyester mooring fiber ropes through the tensile testing of small-scale elements and sub-ropes^15^ emphasized the tensile behavior of nylon and polyester braided ropes, and^16^ investigated the mechanical properties of the high modulus polyethylene (HMPE) and aramid fiber ropes. Some studies proposed special experimental protocols to capture the strength behavior of HMPE^17,18^, polyester, nylon^19^, and aramid^20^ ropes. Subsequently, they used the experimental data as input for rope modelling and validation. The drawn conclusions unanimously alluded that utilizing the well-established experimental datasets and testing methodologies is indispensable for an elaborate prediction of the new fiber ropes' strength properties.

On the other hand, as the experiments are expensive, several studies developed analytical and numerical models to predict the synthetic fiber ropes' response. For instance^21^, outlined computational codes and included them in software (FRM) for predicting the quasi-static tensile response of twisted ropes, whereas FRM was used elsewhere for numerical simulation and validation of ropes performance^22,23^. After that, more rope modelling strategies were supposed, such as the continuum model^21^ and the unified modelling approach^24^. Moreover, 3D finite element models were implemented to accentuate and predict the static tensile response of fiber ropes^25–28^. However, the formulation of the mathematical and numerical models is sophisticated and has impediments. It predominantly requires approximations and a deep understanding of the ropes' ambiguities due to their heterogeneous nature. This causes uncertainties in the experimental, analytical, or numerical approaches for predicting ropes' strength, hampering the progress of ropes applications. The root of the mentioned difficulties is the statistical nature of fiber strength, where most characteristics or properties of fibers can vary within a certain range, and they follow statistical patterns or distributions rather than having fixed, consistent values^29^. This profoundly affects the rope's response and increases the need for statistical models.

In the context of establishing effective experimental datasets, the reliability of any prediction study is unattainable without well documentation and analysis of these datasets. Consequently, it is pivotal to utilize systematic statistical methods to describe the characteristics of fiber ropes and enhance the understanding of their behavior based on experimental records. It is not uncommon in the literature to find studies that applied statistical approaches, such as Weibull statistics^30^ to predict tensile properties of composite materials^31,32^, natural fibers^33,34^, or natural yarns^34^. Conversely, the statistical models for assessing synthetic fiber ropes' tensile strength are far less available. Nevertheless, there have been few endeavors to develop statistical models for the strength of yarns and cables based on yarns' constructional parameters^35^. In addition, regression analysis was implemented to predict yarn-breaking strength and elongation^36,37^. The linear regression showed some limitations due to the nonlinear relationships in the data. However, the artificial neural network (ANN) models can address these limitations unequivocally^29^. The ANN represents a very powerful machine-learning technique that imitates some functions of the human brain and allows learning from examples. The major distinction between the traditional statistical methods and ANN lies in the basic functions that fit the input–output data, where ANN uses simple functions (usually sigmoidal) and combines them in a multilayer nested structure^38^. ANN finds applications in several fields, such as electronics and medical due to the large amount of data, material science, for example, predicting and optimizing tensile properties of polymeric composite materials^39^.

More importantly, ANN has been versatile in textile technology, particularly in engineering the textile yarn's strength properties through building prediction models that outperformed the mathematical and statistical ones. Üreyen and Gürkan 2008^37^ designed ANN models to predict tensile tenacity and elongation of cotton yarn from their fiber properties based on analyzing 180 produced samples. Das et al. 2013^40^ and Doran and Sahin 2020^41^ engineered the manufacturing of cotton yarn with requisite quality by selecting the proper raw materials using the ANN models to map the relation between fiber and yarn properties. Özkan et al. 2014^36^ employed the ANN to investigate the impacts of specific intermingling process parameters on the breaking strength and elongation of polyester yarn. They also concluded that ANN has shown better results than that of linear regression. Razbin et al. 2023^42^ developed an ANN model with efficient and accurate prediction capability to study the influence of structural parameters, such as the initial helical angle of the wrap component and the diameter ratio of components, on Poisson’s ratio of a double core helical auxetic yarn. By using the ANN, no time or effort has to be paid to generate the mathematical equations for the unknown relations, e.g., as required by nonlinear regression^43^. To the authors' knowledge, the application of statistical and predictive techniques based on experiments, for characterizing the strength of synthetic fiber ropes, is limited due to a lack of experimental statistical data on these ropes. Thus, the study is considered as a first contribution.

The current study presents experimental-based statistical models for predicting the tensile properties of synthetic fiber ropes made of polyester, polypropylene, and nylon polymeric fibers based on a well-documented database. The database is compiled following a proposed test protocol that includes tensile tests on fibers, yarns, and ropes as well as differential scanning calorimetry (DSC) tests on the used materials. The research aims to generate experimental statistics that describe the tensile behavior of the available synthetic fiber ropes. These statistics will be utilized to derive simplified stress–strain constitutive laws that are informative enough to be used in practical applications, such as finite element simulation. Finally, prediction models will be developed to predict the simplified constitutive laws of these ropes. We focus, as an onset, on linking the rope structure and some fiber processing parameters with the resultant tensile properties, which allows us to understand the *process-structure–property* relations of the ropes to some extent. In addition, the strategy of this study is expected to monotonously reduce the difficulties and dispersions in developing the mathematical or numerical models of the investigated ropes. It is not intended to make the current study all-encompassing; however, it contributes to minimizing the cost and efforts in designing the synthetic fiber ropes and predicting their tensile properties.

The flowchart in Fig. 1 explains the adopted methodology. It highlights the various applications of synthetic fiber ropes in the practical industry. To establish the dataset of this study, 196 samples of the various ropes were prepared to conduct the tensile tests under two loading conditions. Additionally, to obtain a deeper understanding of the *process-structure–property* of the rope, the thermal history of the fibers was analyzed, and the tensile properties of lower-level components (fiber, yarns) were also tested. After calculating the stress–strain curves for each rope, tri-linear constitutive laws were derived using the piecewise linear approximation technique for both the mean and individual curves. Based on the results of the conducted tests, a final experimental-based dataset was established, containing the predominant thermal and tensile properties of the fibers as well as characterized tensile properties of the ropes, such as breaking strength and elongation. Subsequently, an ANN machine learning model was developed and validated to predict the characteristic points (coordinates) of the tri-linear stress–strain diagrams of the studied ropes.

**Figure 1:**
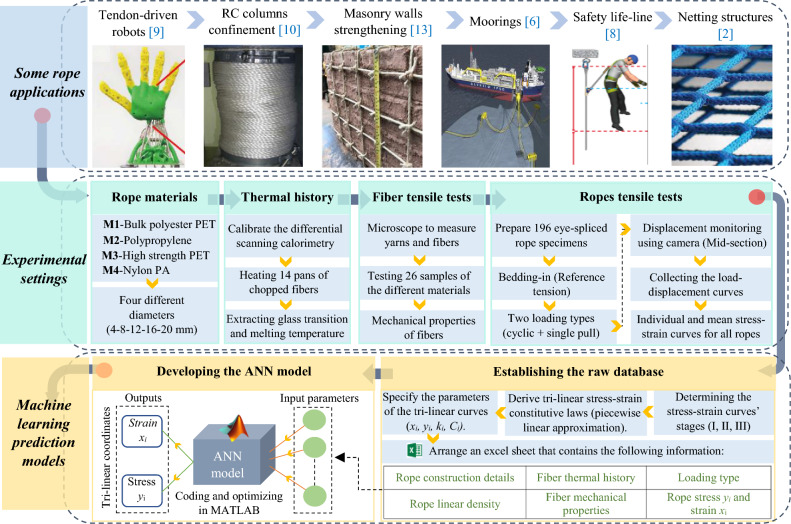
Research methodology and work flowchart.

## Materials and experimental methods

### Rpes main features

**Figure 2:**
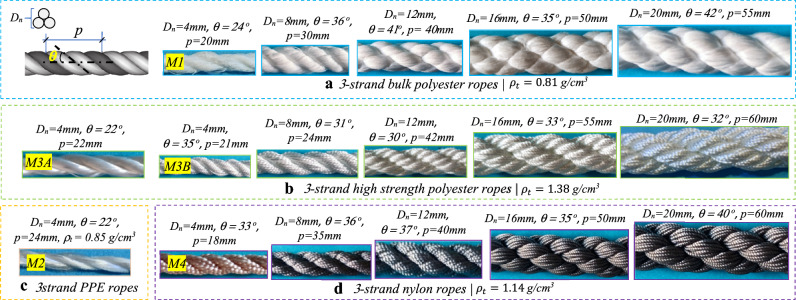
Geometrical parameters of the polyester, polypropylene, and nylon ropes.

**Figure 3:**
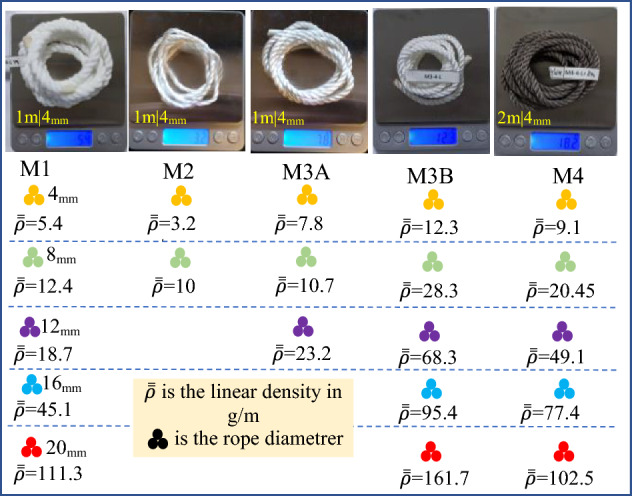
Ropes linear density measurement and results.

The polymeric materials in this study are widely used in rope manufacturing, which are polyester, polypropylene (PPE), and nylon. The synthetic ropes' geometrical parameters and some information provided by the manufacturers Jingchang and Shandong Jinyue textile factories are shown in Fig. 2. The ropes structures are mainly three strands (sub-ropes) twisted and laid together, each consisting of several yarns (strands). The authors selected the diameters and formation patterns of the ropes according to specific criteria, such as easy handling and mounting in the testing machine, controlling the cost and time, and considering that the three-strand laid and twisted is the most widely-used fiber rope structure^2^. As the small-size rope results can be used to estimate the large-size rope response under the same testing conditions^44^, the nominal rope diameter (${D}_{n}$) varies from 4 to 20mm. For a precise interpretation of the characteristic mechanical properties, the rope equivalent cross-section (${A}_{s}$) (fiber area) has been determined by dividing the rope's linear density ($\overline{\overline{\rho }}$) by material density (${\rho }_{t}$), resulting in the rope's solid diameter (${D}_{s}$). For instance, the 4 mm bulk polyester rope has an equivalent diameter of 2.75 mm. This is the first result: a substantial difference can be observed between the effective and the declared diameters. The ropes' linear densities measurement method and results are shown in Fig. 3, where 1 m of each rope type within different diameters is weighted using a high-precision electronic scale. The pitch distance $(p)$ and helix angle ($\theta$) are measured by adapting a prescription addressed in ISO 2307 standard^45^. It should be noted that, in this study, there are two different types of polyester PET ropes, namely, bulk polyester ropes coded as (M1) and high-strength polyester ropes (M3), where the M3 ropes have two fiber types (A, B), as will be revealed by the thermal analysis.

### Laboratory characterization

#### Differential scanning calorimetry (DSC)

**Figure 4:**
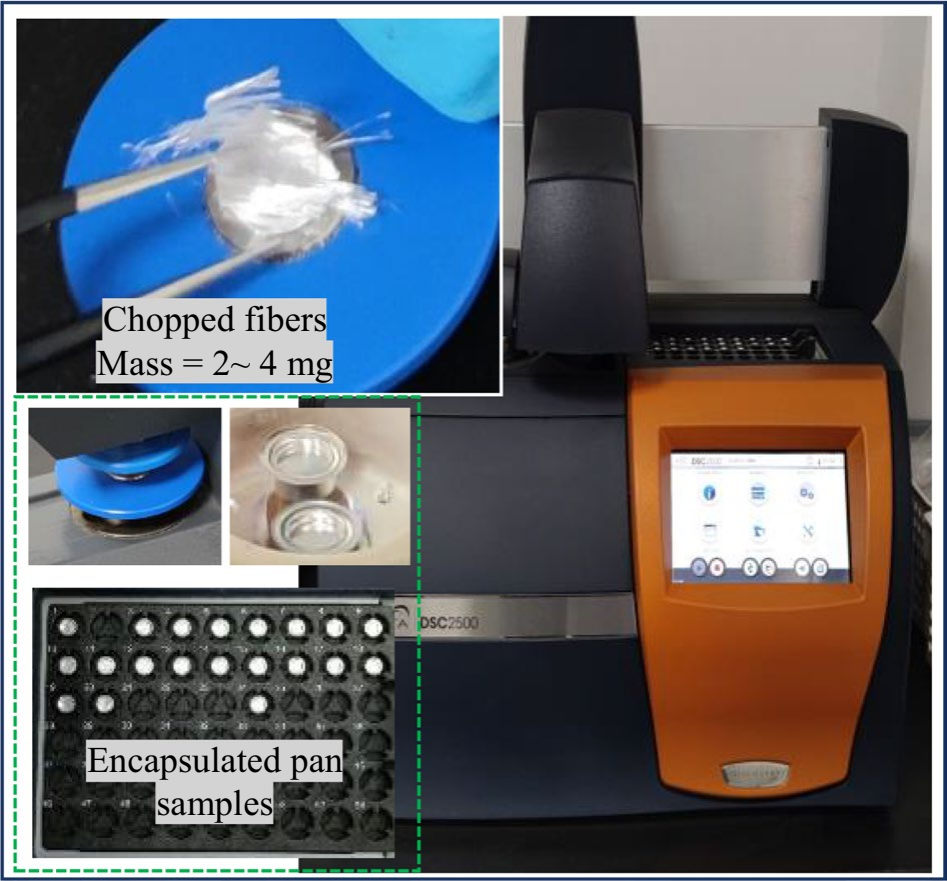
Thermal analysis DSC instrument and chopped fibers.

Thermal analysis (TA) is an effective tool to determine the orientation and mechanical history of fibers or films, which is called structural "fingerprinting"^46^. With this analysis, it is convenient to enhance understanding of the process-structure–property relationship and the knowledge of fiber manufacturing parameters. DSC is an excellent TA technique and gives a global view of the polymer properties and structure ^1^. The DSC aims to monitor the phase transitions (glass transition *T*_*g*_, melting point *T*_*m*_, and crystallization *T*_*c*_), heat capacity *(C*_*p*_), and crystal morphology of the polymeric fibers. These parameters will be employed to further characterize the fibers in this study's statistical and prediction models, where there are divergences in the manufacturers' information. The analysis is performed using the DSC2500 TA instrument and following the ISO 11,357–1 standard^47^. Considering that synthetic fibers are drawn and oriented, the "*free-to-shrink*" method is used to measure the thermal properties of fibers. In this way, the fibers are chopped into small pieces so the fiber's end is free to shrink during heating. Afterward, 14 samples of different materials are weighted using an electronic microbalance; however, the masses vary between 2 and 4* mg* to decrease the sample's temperature gradient and are then encapsulated in a standard ISO pan to ensure thermal contact, as shown in Fig. 4. After loading the sample into the instrument, calibration is done, and the heating rate is set to 10 *°C/min* because the analysis aims to study the first heating result, which provides the desired "*fingerprinting*" of the sample with processing history.

#### Fibers and yarns tensile testing

**Figure 5:**
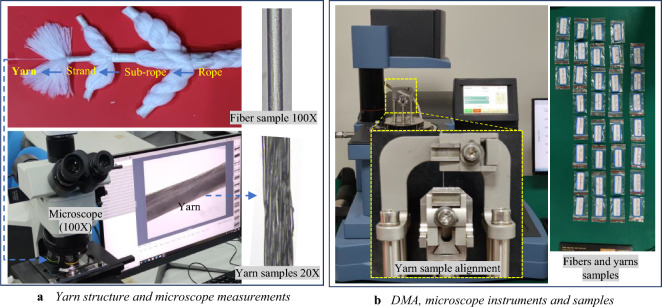
Fibers and yarns tensile characterization tests apparatus and samples.

Fibers or filaments are the essential individual elements of synthetic ropes. Examining the fiber and yarn tensile properties is advantageous because this allows separating the effects of rope construction from the material response. Considering that fiber tensile testing is complex due to the tiny diameters (20 to 50 μm), a small number of single fiber tests is carried out to reinforce understanding of the strength properties of the ropes’ raw material. However, sufficient yarn tests provide fiber properties because they are somewhat less scattered than fiber tests^1^. Consequently, small textile yarns are produced by bundling and interlacing nearly parallel fibers to give some coherence to the yarns' structure, see Fig. 5a. These yarns are very small, in the order of 0.1 to 0.2 mm, where the diameters of fibers and yarns are measured in an electrical microscope with an amplification ratio of 100X by taking the average diameter resulting from different locations along the sample, as shown in Fig. 5a. Afterward, 26 quasi-static tensile tests are performed in a DMA (Dynamic Mechanical Analyzer**)** machine following the recommendations in preparing the samples by ASTM D3822^48^ and ASTM 2256^49^ standards for fiber and yarn tests, respectively, see Fig. 5b. Table 1 lists the microscope measurement results, specimens' lengths, loading speed, and sample sizes for the different materials. Note that, at the start of the tensile tests, it has tacitly been assumed that the fiber is initially straight after applying a pre-tension force to pull out the possible crimp in the specimen. The outcomes from the DMA tests are the load-extension curves taking into consideration removing the unsatisfied results.

**Table 1:** DMA specimens' properties for fibers and yarns quasi-static tensile tests.

Level	Material	Material code	Diameter (*μm*)	Length (*mm*)	Loading speed (*mm/min*)	Pre-tension force (*N*)	Sample size
Fiber (**Yarn**)	Bulk polyester	M1	45 (**110–150**)	8–11 (**9–11**)	**0.3 mm/min** for fibers and **1 mm/min** for yarns	**0.001 N** for fibers and **0.01 N** for yarns	2 (**3**)
	Polypropylene	M2	54 (**150–200**)	8–10 (**9–12**)			1 (**3**)
	High strength polyester	M3A	47 (**125–250**)	8–10 (**8–12**)			1 (**3**)
		M3B	47 (**125–250**)	8–10 (**8–12**)			3 (**3**)
	Nylon	M4	25 (**125–180**)	8–14 (**8–11**)			3 (**4**)

The bold values are for yarn.

### Ropes tensile testing system

#### Specimens preparation

**Figure 6:**
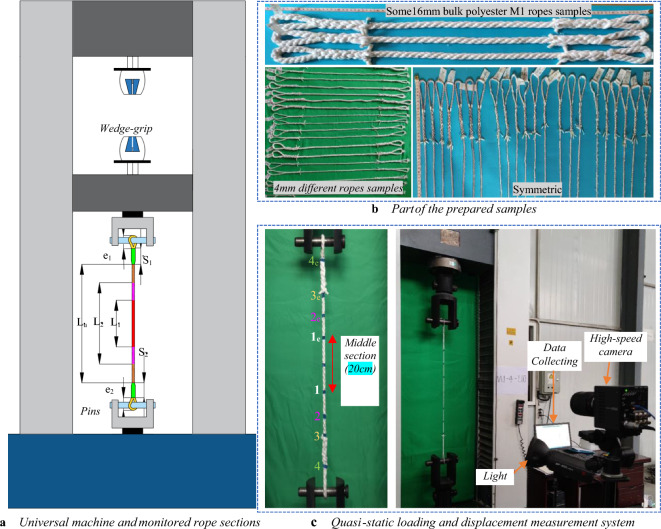
Rope specimens and experimental setup for elongation and load measurement.

As requested, the manufacturers supplied 150 m of different ropes divided into 2 m lengths of each rope piece. ISO 2307^45^ procedures are followed to prepare the specimens and calculate the rope's linear density. The linear density is obtained by measuring the mass per meter of 2 m rope after applying 20% of the reference tension (*T*_*ref*_) in Eq. (1) to pull the slack out of the specimen. Thus, the ropes' linear densities varied from 0.03 g*/cm* to 1.62 g*/cm* due to the differences in ropes' diameters and materials. Intuitively, the fiber rope is as strong as its weakest link, which is the mechanics of termination. Previous research^7,23^ and preliminary tests in this study indicated that an eye splice is the most dependable method of rope termination, giving the optimum tensile performance and preventing end slippage during the tensile tests. The dimensions of the specimens have been derived following^45^, where the total rope length consists of splices lengths (*S*_*1*_, *S*_*2*_), eyes lengths (*e*_*1*_*, e*_*2*_), and the undisturbed gauge length (*L*_*u*_); see Fig. 6a and b. However, *L*_*u*_ is determined to be in the range of [50–60] cm, while the splice length and eye length have been varied into^5–15^ cm and^5–10^ cm, respectively, considering the height limitation of the tensile testing machine. The reasons beyond the diversity in splice and eye lengths are the difficulty in making the splices and the variation in rope diameters. The ropes are prepared 24 h before the test supposing constant temperature and humidity in the laboratory.

1 $${T}_{ref}=1.38\times {({D}_{n})}^{2}$$

where *D*_*n*_ is the nominal diameter in *mm*, and *T*_*ref*_ is the reference tension in newton *N* according to^45^.

#### Tensile test parameters

The pilot experiments, to determine the proper manner of preparing the ropes samples, formed an essential step in the design of the experiment. The preliminary tests have identified the parameters influencing the rope tensile performance. This step aims to reduce the experimental bias by decreasing the sources of hard-to-change variables due to cost and time constraints. For instance, the termination method is chosen to be an eye-splice by alternating and comparing with different mounting ways of the ropes, such as monkey chain, eight loops, timber knot, and normal knot. In addition, the loading type includes two strategies: (1) with a bedding-in process or cyclic loading (CL) followed by a direct pull-to-break, and (2) single pull-to-break (SPB), aiming to enrich the statistical database with the possible rope loading cases. The CL process includes 3 to 5 loading cycles to 20% of the minimum breaking load of the ropes, which helps in molecular reorientation within the fibers and organizing the fibers along the neutral fiber of the ropes^45,50^. In both loading types, a reference tension in Eq. (1) is applied, and a constant loading speed of 250 mm/min is set up for pull-to-break loading^45^. As the universal testing machine in Fig. 6a only provides the piston displacement (pins or wedge-grips ends), the noncontact elongation measurement method is conducted by utilizing a high-speed camera to monitor specific points' movement along the rope, see Fig. 6c. The displacements of the marks on the rope in Fig. 6c are obtained by analyzing the collected videos with *ProAnalyst* video processing software. However, only the strain of the length ${l}_{1-1e}$ is considered. The distance ${l}_{1-1e}$ has been unified for all the ropes to be 20 cm, in order to eliminate the effects of splicing on the rope stiffness and slippage near the termination. For all the tested samples, force–elongation diagrams have been recorded by the data acquisition machine, including the breaking force (*F*_*u*_) and breaking elongation (*∆L*_*u*_). As regards failure, the place where it occurs has been considered satisfactory only at the middle of the ropes or near the splice' ends, but not at nodes.

Two replicate measurements for each combination of *material/diameter/loading type* are chosen from the preliminary rope tests to obtain adequate sample sizes for the tensile tests, resulting in 56 samples. The results are arranged as an instructed database that is statistically analyzed relative to the goal of the final investigation, which aims to estimate the ropes' tensile strength. The dataset features are the materials, diameters, loading types, breaking forces with their standard deviations, and the desired precision to estimate the mean breaking force for each combination. It was clear that the mean forces are different between the materials and diameters, resulting in differences in the standard deviations (*SD*), which ranged between 0.06 and 2 *kN*. For this reason, it is efficient to analyze the relative variations, which means that the sample sizes should be calculated based on the relative precision by using the log-transformed response^51^. The calculation of the sample sizes (*n)* to get an approximate 95% confidence interval for the forces is manifested in applying Eq. (2), which can be found in^51^.

2 $$n=16\times \frac{{({c}_{V})}^{2}}{{(\Delta \mathrm{log}\mu )}^{2}} ; \Delta \mathrm{log}\mu =\mathrm{log}{\mu }_{0}- \mathrm{log}{\mu }_{1}$$

where μ is a specific mean force; *c*_*V*_ is the coefficient of variation of the data, which is independent of combinations and calculated for the entire dataset (preliminary tests) to be *c*_*V*_ = 0.15; $\Delta \mathrm{log}\mu$ represents a difference in the mean log(force) values on the logarithmic scale, which depends on the desired relative precision to estimate the mean force. In this study, the authors assumed that a relative precision of ± 8% to ± 12% is acceptable to estimate the mean breaking forces, which gives $\Delta \mathrm{log}\mu$= 0.16 to 0.24, respectively.

By substituting the previous values in Eq. (2), the sample sizes can be in the range of [14 ≥ *n* ≥ 5] samples for each combination of *material/diameter*, where the loading type's effect was negligible on the scattering of the log(force) values in the logarithmic scale. This range guarantees the confidence placed on the final dataset to be statistically significant in estimating the ropes' tensile properties, in addition to facilitating the conducting of the experiments with effective time and cost. However, each combination's sample size can be estimated separately based on the divergencies in the preliminary tests' results. The planned sample sizes for the combinations of *material/diameter* are shown in Table 2, where *n* is the sum of SPB and CL columns. The total number of rope tensile tests that have been conducted is 220, but the unsatisfactory results were removed from the dataset as proactive and preparedness procedures for data processing.

**Table 2:** Final planned sample sizes (n) for ropes tensile tests.

*D*_*n*_ (mm)	Bulk polyester (M1)			Polypropylene (M2)			High-strength polyester (M3A)			High-strength polyester (M3B)			Nylon (M4)			Total sample sizes
	*SPB*	+	*CL*	*SPB*	+	*CL*	*SPB*	+	*CL*	*SPB*	+	*CL*	*SPB*	+	*CL*	
4	6	+	6	5	+	6	8	+	8	5	+	4	9	+	6	63
8	4	+	4	7	+	5	5	+	2	6	+	3	6	+	3	45
12	3	+	3	6	+	2	5	+	3	3	+	2	8	+	3	38
16	8	+	7	*		*	*		*	7	+	5	5	+	3	35
20	4			*		*	*		*	3	+	1	5	+	2	16
Total	25	+	20	18	+	13	18	+	13	24	+	15	33	+	17	196

*The diameter is unavailable from the manufacturer.

## Experimental results and discussion

### Laboratory characterization

#### Differential scanning calorimetry (DSC)

**Figure 7:**
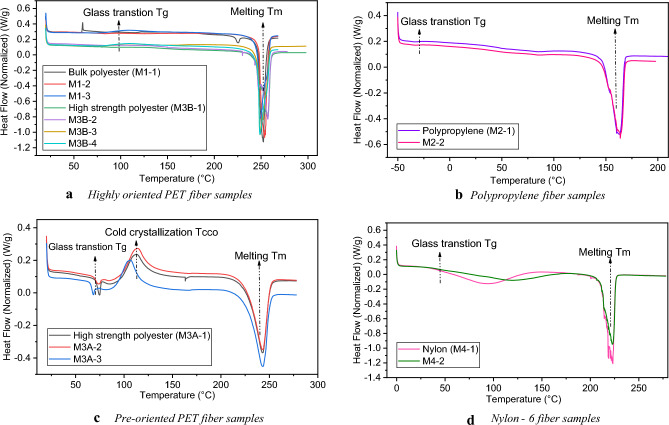
First heating DSC results for the different materials.

Melting experiments provide the desired information about the fiber structure and manufacturing process, which enhances understanding of the *process-structure–property* relationships. Figure 7 illustrates the DSC curves of the first heating round for the different materials in this study. It was evident that the characterization of fibers is obtainable through the interpretation of DSC curves phases and properties, particularly the glass transition temperature (*T*_*g*_), melting temperature (*T*_*m*_), and cold crystallization (*T*_*c*_). Table 3 provides the DSC samples' features and test results with material identification, which agrees with the reported information in the literature^29,46,52^. However, *T*_*g*_ was determined at the midpoint of the first heat capacity jump, and *T*_*m*_ is defined as the highest temperature point of the melting endothermal. Cold crystallization occurs above *T*_*g*_, see Fig. 7c, indicating the peculiar behavior of amorphous polyester for M3A fibers, where the conditions of the fiber spinning speed process affect the kind of these fibers^1^.

**Table 3:** DSC parameters and output for material characterization.

Fiber characterization (material code)	Samples masses (*mg*)	Heating rate (*°C/min*)	Heating range (*°C*)	Average *T*_*g*_ (*°C*)	Average *T*_*m*_ (*°C*)	Average *T*_*c*_ (*°C*)
Highly oriented PET (M1)	2.6, 2.47, 2.68	10	20 to 270	80.92	252.36	–
Polypropylene PP (M2)	2.85, 3.14	10	−50 to 200	−36.43	163.62	–
Pre-oriented PET (M3A)	3.93, 3.59, 4.18	10	20 to 280	69.86	243.14	110.7
Highly oriented PET (M3B)	3.06, 3.54, 3.95, 3.79	10	20 to 270	80.95	252.4	–
Nylon 6 PA (M4)	3.19, 3.76	10	0 to 280	47.55	222.94	–

Therefore, depending on the DSC curves and a previous study^53^, the polyester fibers are categorized into highly oriented yarn HOY (high spinning speed) and preoriented yarn POY (low spinning speed) polyethylene terephthalate PET as shown in Fig. 7a and c, respectively. More specifically, the physical structure "*fingerprinting*" of the PET fibers is described, where the HOY fibers are partially crystalline and highly oriented, resulting in a more uniform macrostructure.

Since the fiber stress concentrates in the oriented region, the taut molecules will break first, decreasing the breaking elongation of the HOY fibers in M1 and M3B ropes^1^. On the other hand, the POY fibers are partially oriented, and their viscosity increases, leading to higher elongation at break^53^. As a result, POY fibers (in M3A ropes) will show the necking phenomenon under tension, where subsequent extension occurs with constant load and involves thinning as the neck moves along the fiber. The results above are considered predictive signs for the elongation properties of polyester rope under tension, which will improve the understanding of the *process-structure–property* relationship. For polypropylene fiber (in M2 ropes), its mechanical properties are determined by chain orientation that is controlled by the spinning and drawing conditions^52^. It can be demonstrated by Fig. 7b that the glass transition is shallow and broad, which indicates that PP fibers are also drawn from semicrystalline polymers. According to our study and^54^, which used PP hot-drawn fibers to study the melting behavior in *free-to-shrink* mode, it is concluded that the PP fibers are drawn with a somewhat high draw ratio (DR = 2.7); however, a high draw ratio gives lower extensions to break and higher tenacity when fiber (yarn) subjected to tension, that is due to the high molecular orientation^52^. Examining the melting behavior of the PP fibers elucidated the fiber's thermal history and gave insight into the fiber structure and its industrial processing, which enhances understanding of the anisotropy of the different fibers' mechanical properties.

Regarding the nylon fibers (in M4 ropes), the DSC heating scan is shown in Fig. 7d. The shape of the DSC curves indicates the presence of lamellar crystals in the **α** and **γ** crystalline forms for the semicrystalline polyamide (PA 6), which is called Nylon-6 with a melting point at about 220 °C based on a previous study^55^. They stated that **α** and **γ** forms are known to coexist in PA 6 with different percentages based on processing conditions; since the stiffness of **α** form is higher than that of **γ** one, the different mixing of these forms imparts different mechanical properties. In our study, consistent with^55^, the heating process revealed that PA 6 exhibits a lower content of **α** phase compared with **γ** one, which intuitively foretells the likelihood of having lower density and stiffness with higher elongation under tension for nylon fibers/ropes. However, the crystalline structure has to be confirmed by performing X-ray analyses, which was not done in the current study and is considered a limitation. As a result, the thermal analysis of the various fibers provides significant veracity for understanding and predicting the *process-structure–property* relationship, increasing the utility of the stress–strain database of the available ropes.

#### Tensile characteristics of fibers and yarns

**Figure 8:**
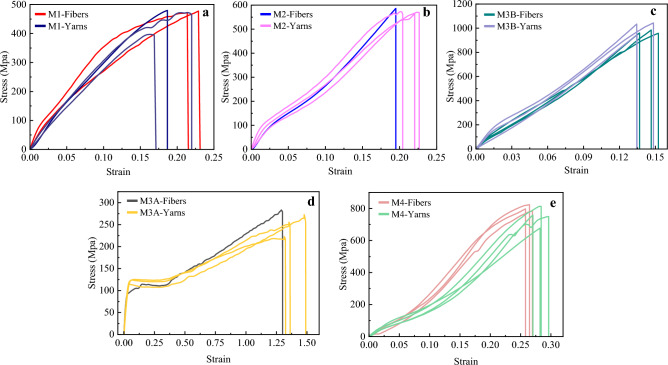
Tensile stress–strain curves of fibers and yarns samples. (**a**) HOY-PET (M1), (**b**) PP (M2), (**c**) HOY high-strength PET (M3B), (**d**) POY-PET (M3A), and (**e**) PA 6 (M4).

Quasi-static tensile tests are carried out on fiber and yarn specimens of the different materials as a further fiber identification process by subjecting the sample to a constant rate of elongation (CRE). Stress–strain relationships are shown in Fig. 8, where Cauchy engineering stress is straightforwardly calculated based on the sample cross-section determined by the microscope. It should be known that the yarns produced as bundles of nearly parallel fibers can describe the fiber properties. This was clear from the similarity and low scattering of fibers and yarn stress–strain curves for each material, in addition to the sudden failure that occurred in all samples, which indicates that the twist effect is negligible. As mentioned in the DSC analysis, the polyester fibers had different thermal histories, which caused different molecular orientations in the fiber structure. The latter point is emphasized by having different tensile deformation behavior for both HOY-PET (M1, M3B) and POY-PET (M3A) samples in Fig. 8a, c and d, respectively. When PET fibers are drawn at a temperature above *T*_*g*_ (hot drawing, with high spinning speed), the shape of the stress–strain curve is what we see in Fig. 8a and c, where deformations somehow took place uniformly along the sample length, and the fibers showed low elongation capability.

On the other hand, if the drawing of PET fibers is done at a temperature below *T*_*g*_ (cold drawing, with low spinning speed), PET will show the necking phenomena under tension^52^, as illustrated in Fig. 8d, where the fiber exhibited high elongation and low strength. For the polypropylene PP samples, the stress–strain response is shown in Fig. 8b, where the effect of the high orientation process was obvious from the curves' shape. Accordingly, the PP fibers are classified as isotropic polypropylene i-PP fibers, which do not experience a wide range of necking and irreversible plastic deformations compared to syndiotactic polypropylene s-PP fibers^56^. It was also clear from the stress–strain curves of nylon-6 fibers in Fig. 8e that the mechanical characteristics of these fibers are influenced by the containment of a predominant percentage of the lamellar crystals in the **γ** phase, where the fibers exhibited a high degree of nonlinearity with lower stiffness and higher flexibility compared with other fibers.

To sum up, the previous results are consistent with the DSC interpretations of the fiber *process-structure–property* relation, where the differences in stress–strain curves' shapes can be attributed to changes in spinning and drawing conditions during the manufacturing of polyester, polyamides, and polypropylene fibers. Subsequently, the mean values of the substantial mechanical properties of fibers are extracted after smoothing and averaging the curves, blurring sharp changes in slope. Therefore, initial Young's modulus (*E*_*0*_) (taken from the linear section at the beginning of the stress–strain curves), fiber breaking elongation (*FBE*), and fiber tensile strength (*FTS*) are organized in Table 4, in addition to the melting *T*_*m*_ and *T*_*g*_ from the DSC results to compare our values with those taken from the literature. However, the published values are typical and have variations, but the actual properties depend on the specific variant of each type of fiber, leading to a range of values^2^. In addition, characterizing the properties of synthetic fibers encounters many difficulties during testing that lead to uncertainties in their properties, for instance, fiber cross-section, length, extension rate, temperature, relative humidity, and the clamping method. The listed values in Table 4 and the stress–strain diagrams in Fig. 8 are only roughly indicative of fiber ropes' mechanical properties in this study, where they will be considered inputs in the prediction models' final dataset. These inputs are postulated to have phenomenal importance in predicting the ropes' mechanical properties based on the *process* relationship with the rope's *structure–property*.

**Table 4:** Main tensile mechanical properties of synthetic fibers compared to those listed in Cordage Institute Charts.

Fiber (material code)	*E*_*0*_ (Gpa)		*FTS* (Mpa)		*FBE* (%)		*T*_*m*_ (*°C*)		*T*_*g*_ (*°C*)	
	Our study	^2^	Our study	^2^	Our study	^2^	Our study	^2^	Our study	^2^
HOY-PET (M1**)**	4.7*	–	460*	–	20*	–	253	258	80.92	70 to 80
PP (M2)	5	6	570	560	21	20	164	165	−36.43	−10 to -30
POY-PET (M3A)	2.2*	–	255*	–	135*	–	244*	–	69.86	65 to 75
HOY-PET high strength (M3B**)**	10.5	15	1138	1130	15	12	252	258	80.95	70 to 80
PA 6 (M4)	3.5	6	812	960	28	20	223	218	47.55	45 to 50

* No reference value in the literature.

### Ropes tensile behavior

#### Tensile test results

**Figure 9:**
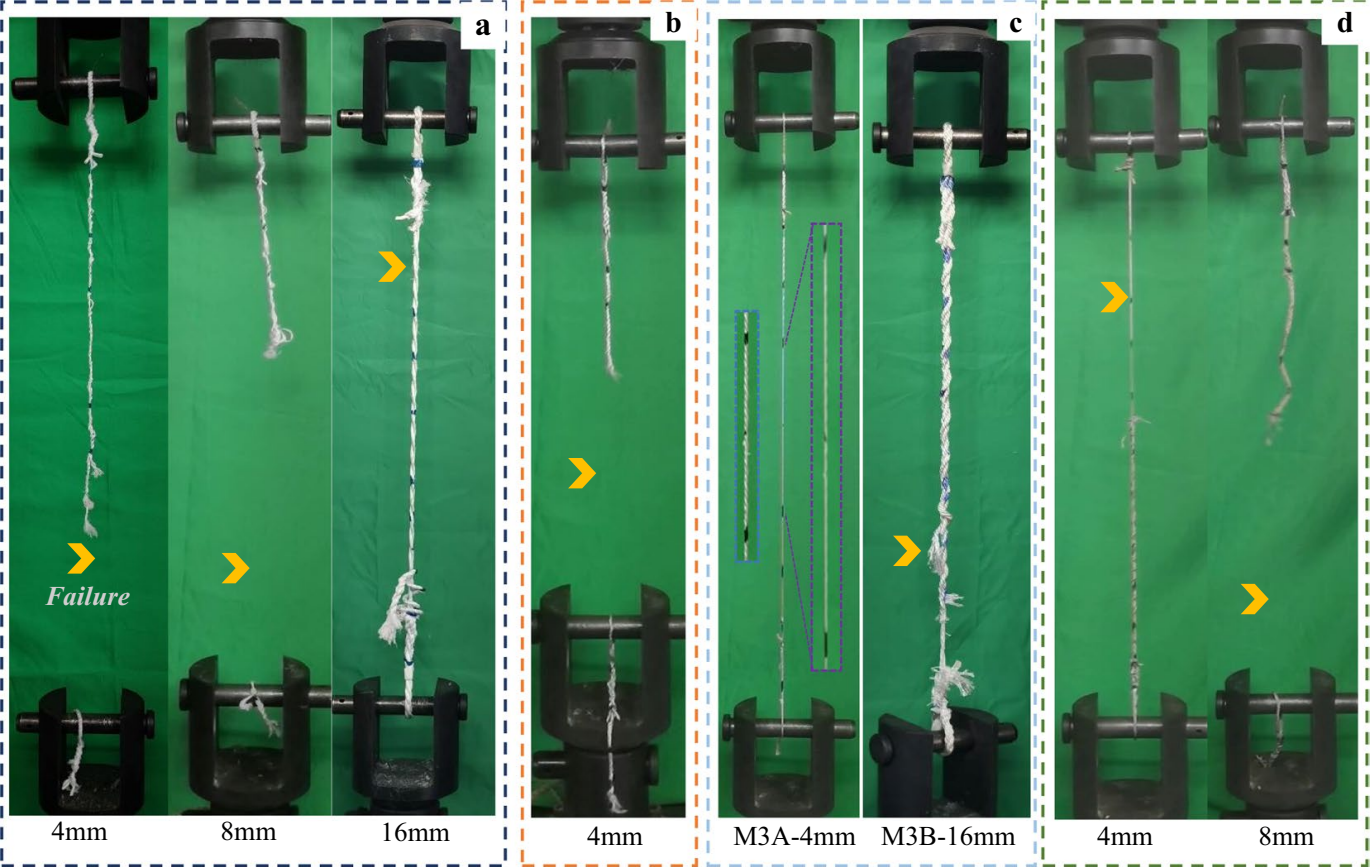
Failure patterns of some (**a**) M1, (**b**) M2, (**c**) M3, and (**d**) M4 ropes at the moment of breaking.

A total of 196 tensile tests were conducted on the different materials and diameters, where the data acquisition machine recorded the load–displacement curves. For more precision in calculating the strain of the rope, the displacement of the rope's middle section was extracted from the high-speed camera video analysis, which also enabled monitoring of the rope's failure modes to accept or reject the failure conitions. The predominant failure modes are shown in Fig. 9 for the different ropes, where the yellow arrow position between the pins remarks the failure location.

As could be observed, with the eye-spliced ends, most of the failures took place along the undisturbed section of the rope (out of splices' legs or eyes), whether in the middle of the rope or near the splice ends, which is acceptable in the splice-structured ropes due to the stress concentration immediately after the end of the buried section of the splice^57^. It also could be seen that some specimens failed suddenly with fragile behavior, and the breakage occurred on the total cross-section of the rope, as in the ropes of M1, M2, and M4 in Fig. 9a, b, and d respectively. At the same time, a progressive partial failure occurred on the bigger diameter ropes as in the case of 16 mm ropes of M1 and M3B, in Fig. 9a and c, respectively. In the latter failure mode, one strand at least fails first, followed by the second and third; however, the failure limit was considered as the failure of the first strand because the complete failure of all strands is rare. As elucidated in Fig. 9c for the 4 mm M3A ropes, there was a quite obvious high extension in the rope and a corresponding reduction in the cross-sectional area due to the necking phenomenon. However, the failure was taken when the necking was spread along the undisturbed gage length of the rope due to the machine's height limitation, where some M3A ropes were not broken.

As mentioned previously, the synthetic fiber ropes have ill-defined cross-sectional areas owing to the complex structure of their fibers' arrangements. Therefore, the nominal cross-section is hard to use for calculating Cauchy stress; instead, the net fiber area (${A}_{s}$) was found based on the rope's linear density ($\overline{\overline{\rho }}$):

$${\text{Stress}}~ = ~{\text{Tension}}~\left( N \right)/A_{s} ~\left( {mm^{2} } \right)~~in~~Mpa\;A_{s} = \overline{{\overline{\rho } }} ~\left( {g/mm} \right)/\rho _{t} ~\left( {g/mm^{3} } \right) \to {\text{Stress}}~ = \left( {{\text{Tension}}/\overline{{\overline{\rho } }} } \right) \times \rho _{t}$$

In textile engineering, it is better to normalize forces as specific stress, namely force divided by mass per unit length (linear density), with N/tex (newtons/tex) being the preferred unit.

$${\text{Specific}}\;{\text{~stress}}~\left( {\frac{N}{{tex}}} \right) = ~{\text{Cauchy}}\;{\text{~stress}}~/\rho _{t} \;{\text{stress }}in\;\frac{N}{{km^{2} }}\;\rho _{t} \;in\;\frac{g}{{km^{3} }}\;,{\text{ and}}\;\left. {1~tex = 1g/km} \right)$$

In this study, the authors chose to display the engineering stress in Mpa since it is assumed that the equivalent cross-sectional area of the rope (A_s_) stays constant during stretching. The option to convert it into specific stress is possible through the previously mentioned relationships. Briefly, the specific stress can be obtained by dividing the stress by a converting factor (CF) shown on the stress axes for each diameter in the stress–strain figures. As a result, the breaking stress ${(f}_{u})$ and breaking strain $({\varepsilon }_{u})$ are found based on Eqs. (3) and (4), respectively.

3 $${f}_{u}={F}_{u}/{\mathrm{A}}_{s}={F}_{u}\times {\uprho }_{\mathrm{t}}/\overline{\overline{\uprho }}$$

4 $${\varepsilon }_{u}={\Delta L}_{u}/{l}_{1-1e}$$

where ${F}_{u}$ is the breaking force, ${l}_{1-1e}$ is the middle section length of the rope, ${\Delta L}_{u}$ is the breaking elongation.

**Figure 10:**
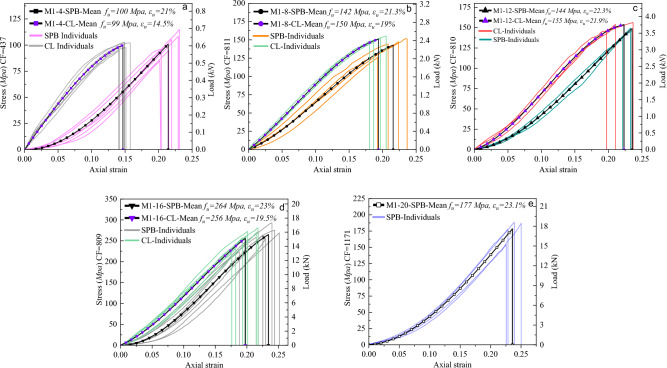
Tensile stress–strain curves of (**a**) 4mm, (**b**) 8mm, (**c**) 12mm, (**d**) 16mm, and (**e**) 20mm of bulk polyester ropes (M1) under both loading types (SPB and CL).

Figure 10 illustrates the relationship between stress and strain in bulk polyester ropes (M1) of various diameters and loading types, after undergoing numerical smoothing to eliminate random data fluctuations. In addition, the equivalent load, the mean tenacity $({f}_{u})$ or breaking stress, and the mean breaking strain $({\varepsilon }_{u})$ are illustrated in the figures for comparison purposes. It was intuitive that increasing the diameter leads to higher breaking forces under both loading types (SPB and CL), which allows the small diameters to be a primal approach to understanding and predicting the behavior of the bigger ropes. Besides, for the SPB samples, the ropes exhibited more extension with increasing diameter, which is reasonable due to the higher amount of irregularity in the molecular chains of fibers requiring more reorientation efforts. Consequently, a concave nonlinear plateau has appeared in the stress–strain curves of these samples resulting in a uniform upward extension. However, with increasing the load, the fibers became more organized, and the curves became perfectly linear before the failure.

The influence of the bedding-in (cyclic loading) process was clear on the shape of the stress–strain curves of the CL samples, where the initial concave portion could be eliminated, and the curve became more linear in this stage in addition to decreasing the breaking strain of the ropes. This phenomenon results from the reorientation and realignment of the fibers in the micro-structure of the rope, leading to a decrease in extensibility but more stabilized mechanical properties, which is consistent with previous research findings^50^. Thus, the usage of the rope, whether new or old, governs the final stress–strain shape that is considered for extracting the rope's mechanical properties. The cyclic loading showed an insignificant effect on the rope's breaking strength, where an unremarkable difference has been determined between the SPB and CL samples. However, CL has attenuated the aberrations in the stress–strain curves to be more similar to the original fibers' responses, as depicted in Fig. 8a of the DMA experiments.

By comparing the strength properties of M1 ropes with their fibers and yarns, it was obvious that the strength of fibers is much higher than that of ropes. This can be interpreted by the construction effect, where the fibers in the rope are not subjected to uniaxial tension due to the twisting effect. As a result, the loading scenario dramatically influences the stress–strain curves profile of M1 ropes, where it removes the geometrical nonlinearity in the rope; however, both CL and SPB scenarios are required to be considered when extracting the ropes' strength properties.

**Figure 11:**
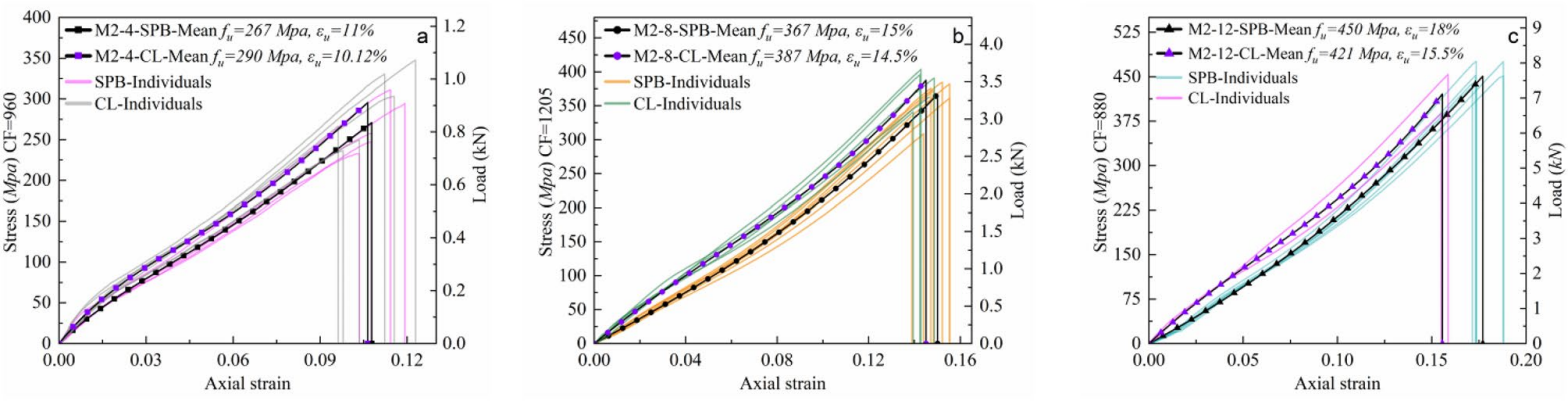
Tensile stress–strain curves of (**a**) 4mm, (**b**) 8mm, (**c**) 12mm of polypropylene ropes (M2) under both loading types (SPB and CL).

For the polypropylene ropes (M2), Fig. 11 shows their stress–strain smoothed curves for the different diameters and loading types. As previously explained in the failure mode of M2 ropes in Fig. 9b and the stress–strain properties of PP fiber in Fig. 8b, the breakage was brittle with low extension, which was indicated on the rope level. As shown in Fig. 11a, there was no considerable difference between the CL and SPB samples regarding the extension, while a slight increase in the tenacity of CL samples was observed. The stress–strain profile changed slightly after the cyclic loading, where the curves started with an elastic linear stage followed by a nonlinear convex plateau with a loss of stiffness due to a reorganization of the entanglements and the unfurling of molecules. Lastly, a work-hardening linear stage appeared after the molecular chain alignment and flew up to fiber rupture, then rope failure. An identical stress–strain profile was previously observed in the fiber tensile tests, as shown in Fig. 8b. These results reveal the compacting nature of the PP fibers inside the M2 ropes, which have a low twisting angle ($\theta = 22$^*o*^*)* compared with other ropes, resulting in the strength similarity between the rope and its fiber level. Moreover, DSC analysis also emphasized that low extensions to break and higher tenacity might exist when PP fiber/yarn is subjected to tension, which was not surprising in the ropes' tensile tests. This is due to the high molecular orientation of PP fibers and high drawing ratio^52^.

**Figure 12:**
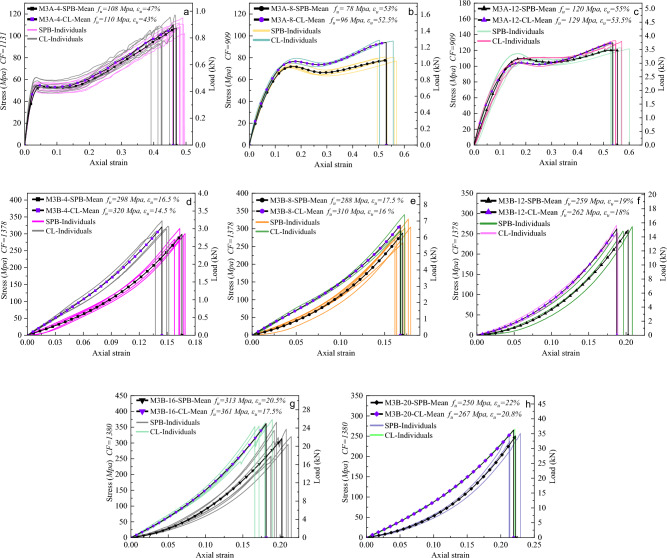
Tensile stress–strain curves of (**a**) 4mm, (**b**) 8mm, (**c**) 12mm of POY polyester ropes (M3A) and (**d**) 4mm, (**e**) 8mm, (**f**) 12mm, (**g**) 16mm, (**h**) 20mm of HOY high-strength polyester ropes (M3B).

Figure 12 illustrates the tensile response of the polyester ropes (M3), which contain high-strength polyester fibers with two different thermal histories. The impression from the rope tests is similar to that from the thermal analysis and fiber tensile tests, where two different stress–strain profiles were recognized for M3A and M3B ropes. Figure 12a–c shows that M3A ropes exhibited high elongation and low strength with ductile behavior (soft and tough). This was speculated by the thermal analysis, where POY-PET fibers were drawn under the glass transition temperature (cold-drawing), resulting in partially oriented fibers with the ability of high elongation under tension. In addition, by recalling the results of fiber tensile tests in Fig. 8d, we could find that the stress–strain models of ropes and fibers are pertinent. Both started with an elastic linear stage followed by yielding with a cold-drawing region, defined as the stress at which measurable strain occurs without a considerable increase in stress, and lastly, with a linear work-hardening stage.

On the other hand, M3B ropes in Fig. 12d–h showed low extension and high tenacity with a nonlinear upward stress–strain curve, which differs from that on the fiber level, where almost a linear behavior was observed, see Fig. 8c. This difference results from the high geometrical nonlinearity that M3B ropes contain and the complexity of the rope's macrostructure. Furthermore, M3B ropes have HOY-PET fibers drawn above the glass transition (hot-drawing) with high orientation, tightening the molecules that break with lower extension than POY-PET fibers. Notably, the tenacity of M3 fibers is more than twice that of corresponding ropes, highlighting the impact of construction effects such as braiding and twisting on the ropes' mechanical properties. Concerning the loading type effect, it was evident that the ropes manifested less extension and higher tenacity after the cyclic loading, which caused a permanent bedding-in strain^50^, revealing the plasticity of the material and the reorientation of the fibrils. It is worth noting that as the diameter increases, there is an increase in failure strain (${\varepsilon }_{u}$) and failure force (${F}_{u}$) but not necessarily an increase in failure strength (${f}_{u}$) due to the stress calculations being based on the solid diameter deduced from the different linear densities of the studied ropes. However, the dependency of synthetic ropes' strength on molecular mass is a well-established phenomenon^1^.

**Figure 13:**
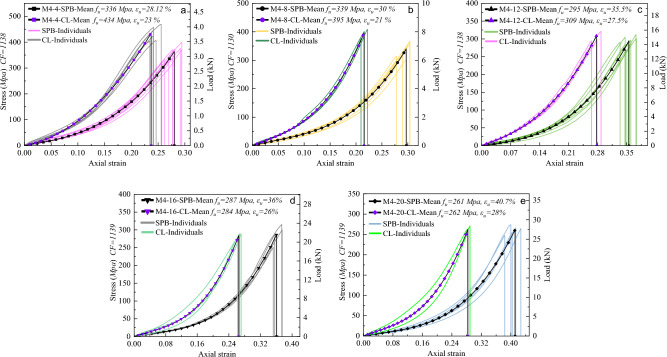
Tensile stress–strain curves of (**a**) 4mm, (**b**) 8mm, (**c**) 12mm, (**d**) 16mm, and (**e**) 20mm of nylon ropes (M4) under both loading types (SPB and CL).

Figure 13 presents the stress–strain and load-strain curves for nylon ropes (M4), where the tension behavior of ropes with five different diameters is examined under two loading types. The static mechanics of nylon ropes showed that they are distinct with a strong nonlinearity, indicating that they are softer than M1, M2, and M3B ropes, which aligns with the findings of a prior study^19^. The stress–strain profile of M4 ropes is characterized by a uniform upward extension with an initial linear portion followed by a nonlinear broad concave curve and, lastly, a linear behavior up until the point of rupture. It is important to note that M4 ropes have a larger reversible strain in the initial linear elastic stage when compared to polyester and polypropylene ropes. Remarkably, the tensile behavior observations from the stress–strain properties of M4 ropes are inextricably related to those obtained from the DSC analysis and fiber tensile tests. The thermal analysis showed that PA 6 fibers would exhibit longer elongation and lower stiffness due to the presence of lamellar crystals **γ** crystalline form for the semicrystalline polyamides. This was demonstrated on the fiber level, where the shape of the stress–strain curve in Fig. 8e and the *FBE* value in Table 4 showed the high elongation and nonlinear behavior of PA 6 fibers. Furthermore, the type of loading had a discernible effect on the tensile response of M4 ropes, where the failure strain of the CL samples was notably decreased by 10% on average compared to SPB samples. It should be noted that there was only a slight increase in the failure stress (tenacity) of CL samples compared to SPB samples. This was found to be the case for the majority of the ropes, which unanimously pointed out that the loading protocol of synthetic fiber ropes affects the failure strain but has a minor impact on the failure stress. Furthermore, increasing the diameter of fiber ropes led to an increase in failure strain and failure force. However, the higher diameter does not always mean a higher failure strength (stress), where ropes with higher diameters may contain more voids than those with small diameters.

According to the inferences described above, characterizing the mechanical properties of synthetic fiber ropes is strongly correlated with the thermal history and tensile properties of fiber/yarn components. Therefore, accurately forecasting the tensile behavior of synthetic fiber ropes necessitates a comprehensive comprehension of the complexities of the *process-structure–property* relationship.

#### Tri-linear stress–strain simplified models

Characterizing the mechanical behavior of the synthetic fibrous elements requires the creation of material models that describe at least the relationship between deformation and stress (phenomenological models). More complex constitutive models are available in the literature to predict the different phenomena of polymeric materials, such as micromechanical models that use knowledge about the material's microstructure as the basis for the model^1,58^. However, it is difficult to develop purely micromechanical models due to the complexity of the deformation characteristics of the molecular microstructure of fibers; thus, there is typically a limited set of constitutive models in the FE software that is appropriate for predicting the polymeric elements' behavior. As synthetic fiber ropes are widely used in engineering applications, there is an imperative need to derive simplified and user-friendly stress–strain models that are, for example, suitable to be used by numerical analysis. It should be known that the mean curves in Figs. 10, 11, 12 and 13 are represented by three-order polynomial functions using the nonlinear fitting technique. This section endeavors to schematize the nonlinear stress–strain responses of the different ropes through a tri-linear constitutive law.

**Figure 14:**
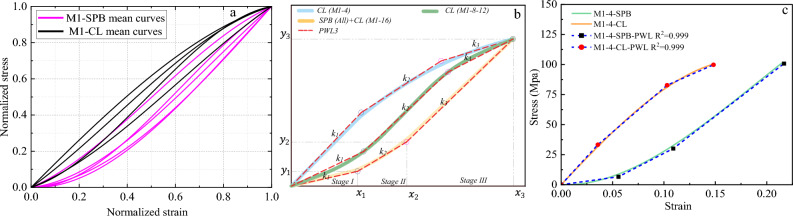
The stress–strain material model of M1 ropes. (**a**) Normalized mean stress–strain for all diameters, (**b**) Tri-linear approximation (schematic view), (**c**) Tri-linear stress–strain scheme with constitutive equations for 4mm ropes.

**Figure 15:**
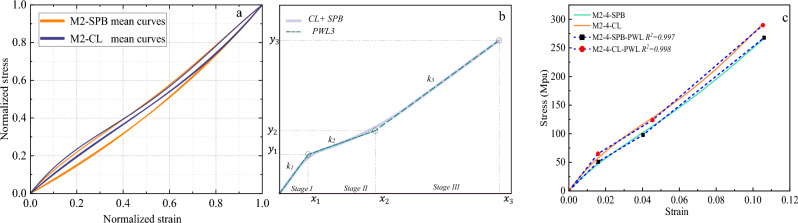
The stress–strain material model of M2 ropes. (**a**) Normalized mean stress–strain for all diameters, (**b**) Tri-linear approximation (schematic view), and (**c**) Tri-linear stress–strain scheme with constitutive equations for 4mm ropes.

**Figure 16:**
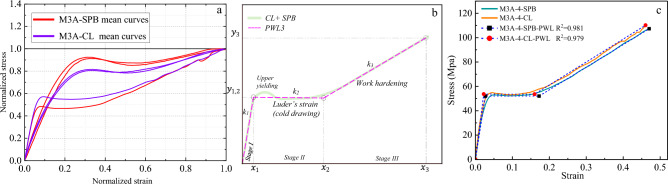
The stress–strain material model of M3A ropes. (**a**) Normalized mean stress–strain for all diameters, (**b**) Tri-linear approximation (schematic view), and (**c**) Tri-linear stress–strain scheme with constitutive equations for 4mm ropes.

**Figure 17:**
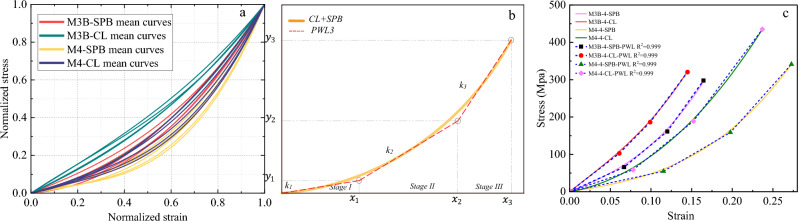
The stress–strain material model of M3B and M4 ropes. (**a**) Normalized mean stress–strain for all diameters, (**b**) Tri-linear approximation (schematic view), and (**c**) Tri-linear stress–strain scheme with constitutive equations for 4mm ropes.

Continuous piecewise linear approximation functions (PWL) have been formulated based on the appropriate convexity/concavity properties of the nonlinear stress–strain curves to simplify them. PWL functions are usually used to develop a programming approximation to a general nonlinear term in the objective function^58^, which is the stress–strain behavior in our case. The first step was to determine what are called "breakpoints" for each stress–strain curve, and the second step involved introducing variables that allow us to formulate PWL functions using linear relations. From Figs. 10, 11, 12 and 13, we could parameterize the stress–strain curves by common characteristic points (breakpoints for PWL functions) that confine three stages of the rope's tensile behavior. *Stage I* represents the linear response of the rope, while *Stage II* reflects the realignment of molecular chains of fibers. This behavior was often nonlinear (convex or concave) as in most cases, but in the case of M3A ropes, it exhibits continuous yielding. *Stage III* is a nearly linear stage that precedes failure, such as the work-hardening stage in M2 and M3A ropes. The linear approximation approach is illustrated in Figs. 14, 15, 16 and 17, and the following Eq. (5) describes the constitutive law for each stage.

5 $$\begin{array}{*{20}c} {{\text{The}}\;{\text{complete}}\;{\text{continuous}}} \\ {\;{\text{piecewise}}\;{\text{linear}}\;{\text{approxiamtion}}} \\ {{\text{unction}}\;f\left( x \right)} \\ \end{array} \;{\text{f}}\left\{ {\begin{array}{*{20}c} {{\text{Stage}}~I:~~f\left( x \right) = k_{1} \times x + C_{1} ~~~~for~~~x < x_{1} ~and~C_{1} = 0} \\ {{\text{Stage}}~II:~~f\left( x \right) = ~k_{2} \times x + ~C_{2} ~~for~~x_{1} < x < x_{2} } \\ {{\text{Stage~}}III:~f\left( x \right) = k_{3} \times x + ~C_{3} ~~~for~~~x_{2} < x < x_{3} } \\ \end{array} } \right\}$$

where *k*_*i*_ is the slope of the line segment of each stage, *x*_*i*_* is* the breakpoints coordinate, and *C*_*i*_ is the *f(x)*-intercept.

The *f(x)* functions have been derived for the mean curves of each *material/diameter/loading type* combination, as shown in Figs. 14, 15, 16 and 17. The normalization principle was employed to clarify the stress–strain shape patterns of each combination, while a schematic view of the tri-linear PWL functions is shown. The approximation of 4 mm ropes is represented in the figures (as in Fig. 14c), where the branches of the tri-linear law are obtained as the ones that better fit the three stages of the stress–strain curve resulting in the highest value of the determination coefficient (*R*^*2*^). Nevertheless, the values of the parameters *k*_*i*_, *x*_*i,*_ and* C*_*i*_ in Eq. (3) have been determined and listed in Table A1 in the Appendix for all the mean stress–strain curves of the various combinations. Table A1 also includes the *y*_*i*_ = *f(x*_*i*_*)* values, where the physical interpretation of *y*_*i*_ and *x*_*i*_ is the stress $\sigma$ and strain $\Delta L$ at the breakpoints, respectively. The parameter *k*_*1*_ refers to Young's modulus of elasticity in linear *stage I*. On the other hand, *k*_*2*_ and *k*_*3*_ are the secant modulus (stiffness) of *Stage II* and *Stage III* and are calculated between (*x*_*1*_*, x*_*2*_) and (*x*_*2*_*, x*_*3*_), respectively. The secant modulus is meaningful in comparing the stiffness of the different stress–strain stages and anticipating the material behavior under different loading types^1^. It is noteworthy that this study not only calculated the coordinates (*x*_*i*_*, y*_*i*_) for the mean curves of the tensile tests but also for the 196 individual rope samples that will be included in the database for the prediction analysis.

## Developing the ANN models

Two modelling strategies can be used to decipher the vagueness of the *process-structure–property* relation of the studied ropes. (1) The traditional modelling methods, including the physics laws and computer simulation. (2) Empirical modelling methods based on statistical equations. There are various approaches for empirical model building (EMB) in the textile field that rely on experimental data, aiming to find a relationship between output variables *y*_*i*_ (response) and input independent variables *x*_*i*_ (predictor). The methods of EMB are classified into three broad categories^38^; (*I*) linear statistical methods, (*II*) nonlinear multivariate statistical methods, and (*III*) neural networks, where separate nonlinear models are applied. The selection of the suitable method is primarily influenced by the data properties and the user's subjective interpretation and whether classification or regression issue needs to be discussed; however, each option has its pros and cons. The appeal of neural networks in the field of textiles lies in their universal approximation, parallel processing, and recurrent dynamic modelling, but the models built by neural networks are usually "black box" and require a large amount of training. Due to the statistical nature of the data in the current study, where unavoidable randomization in the output or input variables might occur, an artificial neural network (ANN) modelling method is supposed for EMB that delivers predictive insights into the ropes' mechanical properties.

### Data acquisition and structure

As previously mentioned, the experimental dataset was established based on a well-calculated sample size, where a statistical analysis was performed and yielded the descriptive statistics of the tested ropes shown in Table 2. The sample size can be considered a good representative of the population; the input features also covered a wide range of values with enough frequency and zero outliers. Therefore, the quality and sufficiency of the dataset to generate the ANN models is guaranteed with a relative precision explained earlier in Eq. (2) and a sample size of 196 rope tests. Regarding selecting the input variables, lot-wise data about fiber properties and corresponding ropes properties were obtained, which are relevant features (11 input parameters) meaningful for predicting the desired outputs (3 measurements) of this study. The fiber properties were; polymer Poisson’s ratio (*ɥ*), material density $({\rho }_{t}$), fiber tensile strength (*FTS*), fiber breaking elongation (*FBE*), fiber Young’s modulus (*E*_*0*_), and melting temperature (*T*_*m*_). The rope noted properties were; rope diameter (*D*_*n*_), helix angle ($\theta$), pitch distance $(p)$, and the rope linear density ($\overline{\overline{\rho }}$). Furthermore, the loading type was taken as an input feature in terms of testing conditions, whether *SPB* or *CL*. The ANN outputs are the results collected from the tri-linear stress–strain curves that contain strain (*x*_*i*_) and stress (*y*_*i*_) coordinates of the corresponding ropes.

### Settings and architecture of the ANN model

**Figure 18:**
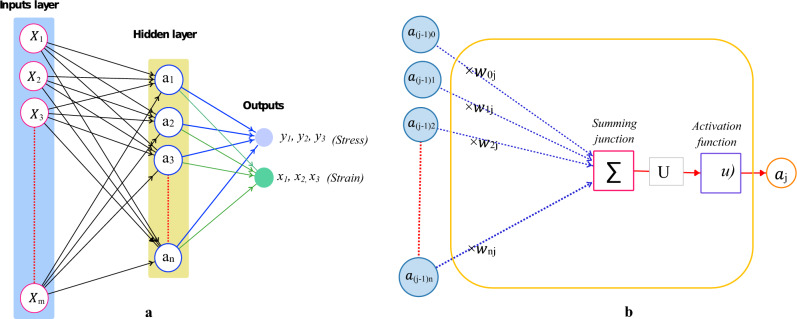
Artificial neuron network. (**a**) Network architecture; (**b**) The mathematical representation of an artificial neuron.

The ANN models are designed as mathematical models that simulate one or more outputs as a complicated nonlinear function of different input variables. Thus, the architecture of the ANN models is constructed by defining three entities; interconnections, transfer functions, and learning rules. The multilayer feed-forward network is employed in this study as an interconnection pattern, which consists of an input layer, output layer, and hider layers. The neurons of the hider layer can detect the underlying characteristics of the input patterns, which are transmitted to the output layer to find the output pattern utilizing output neurons. The number of neurons and hidden layers (hyperparameters) should be optimized to avoid underfitting or overfitting the model. In this study, the number of neurons in the hidden layer is optimized, while the number of the hidden layers is fixed at one due to increasing the computation time exponentially with increasing the number of hidden layers^38^, see Fig. 18a.

The multilayer feed-forward network is trained with the back-propagation (BP) learning algorithm for the determination of the weights. In the BP algorithm, the input data is exchanged between the neurons of the different layers to produce the output. The net weighted input of each neuron is calculated based on the formula in Eq. (6) and then transmitted through an activation function. Subsequently, by comparing

6 $$U = \mathop \sum \limits_{i = 1}^{m} X_{i} \times w_{ji} + b \times w_{j0}$$

the actual outputs with the predicted outputs, the output error is computed and propagated in reverse to fine-tune the individual weights (explicit parameters). The network’s performance is measured by root mean squared error (RMSE), and the weight is updated till the RMSE is reduced to a predefined level. Figure 18b depicts a mathematical model of the artificial neuron.

where *m* represents the number of inputs; ${X}_{i}$ and ${w}_{ji}$ are input signal and weight for the *ith* neuron in the *jth* layer, respectively; *b* and ${w}_{j0}$ are the bias and its weight, respectively.

To optimize the explicit parameters and hyperparameters of the ANN models, the dataset is split into training, validation, and testing sets. The K-fold (fivefold) cross-validation method is used to divide the data into the aforementioned sets and to avoid the bias that could be caused by using fixed sets. The number of neurons in the hidden layer is optimized by generating several ANN models for the simplified stress–strain curves of the studied ropes with different neuron numbers in the hidden layer. Subsequently, the resulting ANN models are evaluated by comparing the following error measures of the models:

7 $${\text{Root Mean Square Error }}\left( {{\text{RMSE}}} \right):\;RMSE = \sqrt {\frac{{\mathop \sum \nolimits_{i = 1}^{n} \left( {\hat{y}_{i} - y_{i} } \right)^{2} }}{n}}$$

8 $${\text{Mean Absolute Error }}\left( {{\text{MAE}}} \right):\;MAE = \frac{{\mathop \sum \nolimits_{i = 1}^{n} \left| {\hat{y}_{i} - y_{i} } \right|}}{n}$$

9 $${\text{Coefficient of Determination }}\left( {{\text{R}}^{{2}} } \right):R^{2} = 1 - \frac{{\mathop \sum \nolimits_{i = 1}^{n} \left( {\hat{y}_{i} - y_{i} } \right)^{2} }}{{\mathop \sum \nolimits_{i = 1}^{n} \left( {y_{i} - \overline{y}} \right)^{2}}}$$

where: ${y}_{i}$, ${\widehat{y}}_{i}$ is the actual, predicted $y$ value of the observation $i$, respectively; $\overline{y }$ is the mean of $y$.

To generate the ANN models and optimize the explicit and hyper parameters, a MATLAB script and associated functions were used to codify the process. Min–Max Scaling (Normalization) was employed to transfer the input variables to confirm that they were on a comparable scale, which facilitated obtaining an efficient parameter optimization of the ANN models. The MATLAB built-in functions for generating the ANN models were applied using the specific settings shown in Table 5. This table contains comprehensive descriptive information about the functions and methods used to generate and optimize the ANN models.

**Table 5:** Full information about the developed ANN models.

Network Architecture	Layers		
	Input	Hidden	Output
Layers No	1-layer	1-layer	1-layer
Neurons No	11-Neurons	1 to 30 Neurons (It will be optimized)	3-Neuron
Connection pattern		Multilayer Feed-Forward Network	

Activation functions	Identity function $\psi (u)\, = \,u$	Hyperbolic Tangent Sigmoid function $\psi (u) = \frac{{e^{u} - e^{{ - u}} }}{{e^{u} + e^{{ - u}} }}$	Identity function $\psi (u)\, = \,u$
Training algorithm	Levenberg–Marquardt Backpropagation		
Data splitting	fivefold cross validation (70% training + 15% Validation + 15% Testing)		
Cost function	Root Mean Squared Error (RMSE)		
Addressing nominal variables	Dummy parameters are generated for the nominal input variables		

### Selection of the optimum ANN model

**Figure 19:**
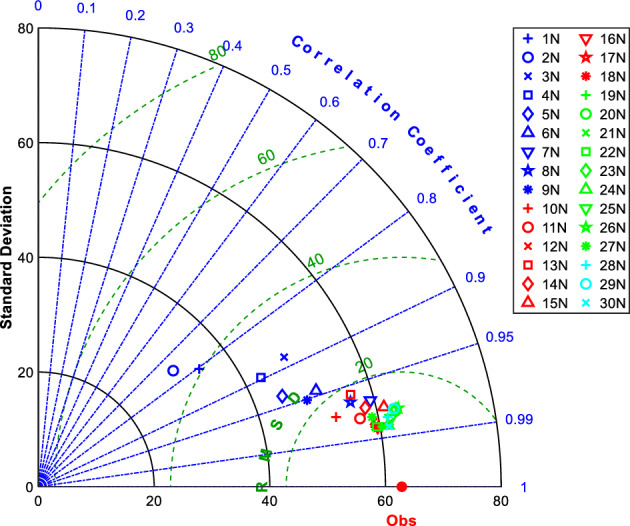
Taylor diagram of the developed models for a stress testing set.

A total of 30 ANN models have been designed and built to predict the stress–strain characteristics of the synthetic fiber ropes. To asses these models’ performance, each of them is evaluated based on three criteria, namely RMSE, R^2^, and MAE error measures. It should be noted that ANN models of predicting the stress values (*y*_*i*_) were running separately from that of strain (*x*_*i*_); however, they were conducted with the same settings. Therefore, three outputs were obtained for each model as shown in Fig. 18a. After the optimization process of the network parameters, the optimum number of neurons that gave the best performance for both stress and strain models was 19 neurons in terms of R^2^ and RMSE error measures for the three outputs of each model. For instance, in the testing set of the ANN model with 19 neurons, R^2^ = 0.9, 0.94, 0.97 for *y*_*1*_, *y*_*2,*_* y*_*3*_ stress coordinates and R^2^ = 0.91, 0.96, 0.975 for *x*_*1*_, *x*_*2,*_* x*_*3*_ strain coordinates; respectively. The model with 19 neurons could provide a higher performance capacity compared to the other predictive models for estimating the tri-linear stress–strain curve coordinates. The Tylor diagram of the stress testing set in Fig. 19 confirms the previous result, demonstrating how the three complementary model performance statistics (RMSE, standard deviation, and correlation coefficient R) vary simultaneously. It identified that the 19N (** +**) model has the lowest RMSE, the lowest standard deviation, and the highest R; thus, it is the best model for reproducing (predicting) certain features of the observations. The same efficiency was in the strain model, leading to accepting the 19N network as the final solution.

**Figure 20:**
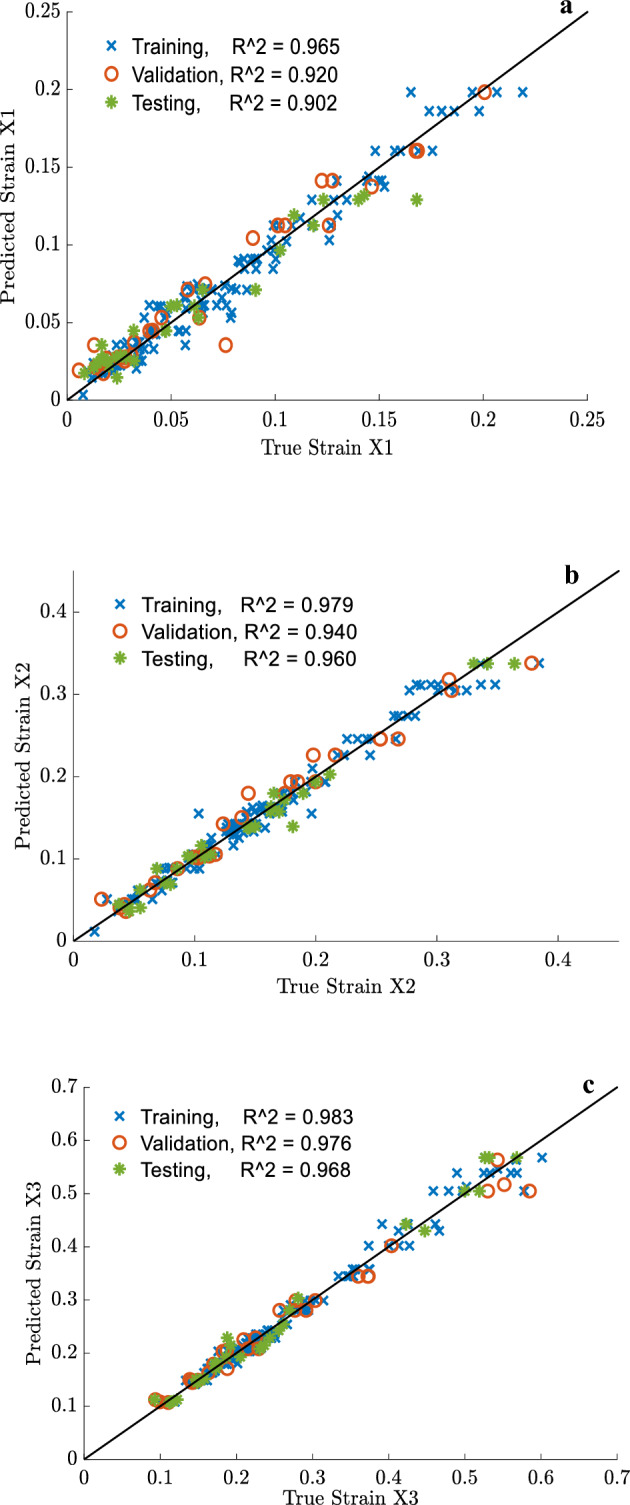
Regression plot of the optimum ANN model for selected training, validation, and testing sets of (**a**) x_1_, (**b**) x_2,_ (**c**) x_3_ simplified strain coordinates.

**Figure 21:**
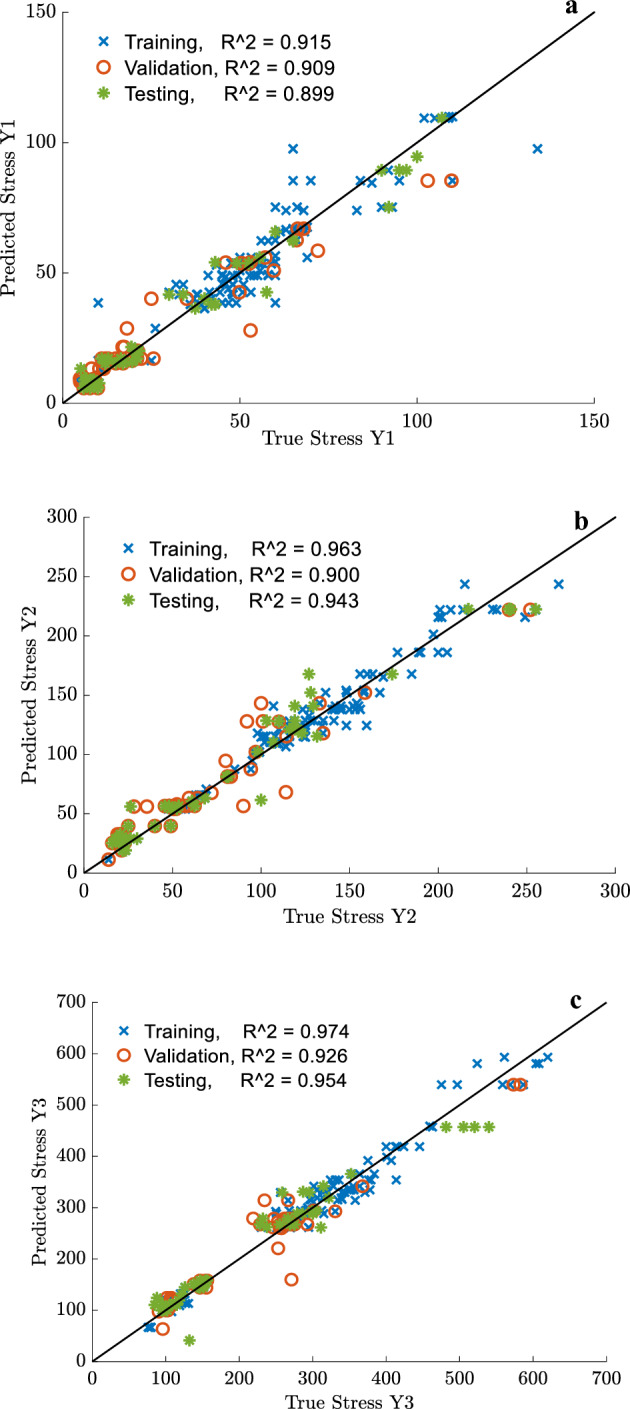
Regression plot of the optimum ANN model for selected training, validation, and testing sets of (**a**) y_1_, (**b**) y_2,_ (**c**) y_3_ simplified stress coordinates.

For further performance evaluation of the developed ANN stress and strain models, regression plots were created. These plots provide a visualization of the relationship between the true (experimental) values and predicted values calculated by the optimum ANN models. Figures 20 and 21 show the regression results of the selected training, validation, and testing sets of the strain and stress coordinates, respectively. The R^2^ values of the testing set for *x*_*1*_, *x*_*2,*_ and *x*_*3*_ are 90.2%, 96%, and 96.8%, respectively. Similarly, the R^2^ values of the testing set for y_*1*_, *y*_*2,*_ and *y*_*3*_ are 89.9, 94.3, and 95.3, respectively. The maximum error measure in evaluating the failure strain and failure stress coordinates was 3.2% and 4.7%. The error is much lower than the maximum acceptable error that the authors selected when calculating the relative precision of the sample size to predict the failure stress and strain, which was 12%. Nonetheless, the precision in projecting the maximum limit (*x*_*1*_*, y*_*1*_) of the stress–strain tri-linear curves for *stage I* is the least accurate compared to *stages II* and *III*, although the error remains under 12%.

Consequently, the ANN model of 19 neurons can be considered an adequate model in estimating the simplified stress–strain constitutive laws of the studied ropes. The model can predict the tri-linear stress–strain curves as a function of several processes, structures, and property parameters of the available synthetic fiber ropes such as *ɥ*, ${\rho }_{t}$, *FTS*, *FBE*, *E*_*0*_, *T*_*m*_, *D*_*n*_, $\theta$, $p$, $\overline{\overline{\rho }}$, and loading type. The selected inputs could generate a reliable ANN model that was built based on a well-documented database with acceptable quality and quantity. As a result, this model can be deployed for use in predicting the tensile properties of synthetic fiber ropes taking into consideration the fiber thermal history, rope construction properties, and loading conditions. Of particular interest in various applications of the ropes are the failure strength and strain, which were accurately forecasted by the prediction model. The developed ANN model is a potent tool for modeling the complex non-linear *process-structure–property* relationship of the available ropes; however, it cannot give a theoretical insight into the mechanics of the relationship between the parameters due to the black box nature of the network. In addition, the model's ability to provide a reliable prediction out of the studied range of data is limited, such as a diameter much larger than 20 mm; however, the neural network can be easily updated with both old and new data. Furthermore, the efficiency of the generated ANN models relies solely on the data collection conditions, data preprocessing, skills in conducting tensile tests on fibers and ropes, and some uncontrollable factors related to the manufacturing process. It should also be considered that the tri-linear behavior is not the only type of behavior that the material exhibits, so it is important to carefully incorporate other material behaviors in the ANN model. While the combinations of material-diameter-loading conditions used in the current study were sufficient for the ANN models to be used in practical work, further research is still required to discover better approximations for the different stress–strain patterns that synthetic fiber ropes may exhibit. Moreover, understanding which structures can provide specific mechanical properties is of interest from an industrial point of view. However, this relation is not straightforward, and using ANN models to establish a reverse relation may not be feasible. Thus, a combination of a trained neural network with an optimization technique such as a genetic algorithm (GA), to explore optimal structures of ropes or optimal fiber manufacturing, is an interesting future research.

## Conclusion

The current study investigated the tensile characterization of synthetic fiber ropes made of polyester, polypropylene, and nylon polymeric fibers. Based on the test results, an experimental dataset was established, containing various parameters of material properties, rope construction, fiber processing, and rope tensile responses. Subsequently, ANN models were developed and optimized using MATLAB to predict the tri-linear stress–strain profiles of the studied ropes. Additionally, tensile testing on fibers and yarns was advantageous because it allowed us to separate the effects of rope construction from the material response. Furthermore, fiber tensile tests reinforced the knowledge obtained from the DSC analysis of the studied fibers, showing consistency between the tensile behavior of the different fibers and their thermal history. Consequently, the results of DSC and fiber tensile tests were considered to be of significant importance in predicting the mechanical properties of the ropes.

In summary, the following observations summarize the general findings from various tensile tests on the ropes under two loading conditions:

- Eye-spliced termination guarantees the correct failure pattern of the rope.
- Small diameters play a crucial role in predicting and understanding the behavior of larger ropes.
- A higher amount of irregularity in the molecular chains of fibers requires more reorientation efforts.

Cyclic loading attenuates aberrations in the stress–strain curve, making it similar to the original fibers' responses. However, the loading protocol influences the failure strain, with a minor impact on the failure stress. The geometrical nonlinearity inherent in ropes can be reduced through cyclic loading.

- The strength (tenacity) of ropes is, in most cases, lower than that of their fibers. This can be attributed to the construction effect, where the fibers in the rope are not subjected to uniaxial tension.
- The stress–strain tensile response of the fiber ropes can be characterized into three stages: Stage I represents the linear response of the rope, Stage II reflects the realignment of molecular chains of fibers, and Stage III is a nearly linear stage that precedes failure. These stages enabled the simplification of the stress–strain nonlinear diagrams into tri-linear stress–strain constitutive laws for the different ropes.
- By connecting rope properties and fiber processing parameters, this research contributed to understanding the process-structure–property relationship of the available ropes.

The prediction models featured the inclusion of certain fiber process parameters, tensile properties of fibers, rope construction properties, and tensile properties of the ropes extracted from 196 individual rope tensile tests. Evaluation of the ANN models using regression plots revealed their ability to accurately predict the failure strength and strain coordinates at Stage III of the tri-linear stress–strain curve, with an error of approximately 5%. However, they demonstrated less accuracy in predicting the Stage I and II coordinates. Nevertheless, the black-box nature of the ANN limits its ability to provide theoretical insight into the mechanics of the relationship between these parameters. Therefore, further research is needed to discover better approximations for different stress–strain patterns that synthetic fiber ropes may exhibit and to understand which rope structures can provide specific desired mechanical properties. In conclusion, this study aims to reduce the cost and effort required in designing synthetic fiber ropes and predicting their tensile properties while contributing valuable insights to the practical industry.

## Data availability

The database of tensile tests will be available by the authors on request. In addition, ANN model code will also be provided with the database.

### Supplementary Information

Supplementary Information.
