# Supplementary material for: Experimental-based statistical models for the tensile characterization of synthetic fiber ropes: a machine learning approach
Source: Sci Rep. 2023 Oct 18;13:17768. doi: 10.1038/s41598-023-44816-x (PMC10584856; doi:10.1038/s41598-023-44816-x)
Supplement: Supplementary file 1 — Supplementary Information. [file 41598_2023_44816_MOESM1_ESM.docx]

**Appendix A**

**Table A1** Piecewise linear approximation parameters of the average tri-linear stress-strain curves of different ropes

| Rope Material | D [mm] | Loading Type | **X1** | **X2** | **X3** | **Y1 [Mpa]** | **Y2 [Mpa]** | **Y3 [Mpa]** | k1 | k2 | k3 | C2 | C3 |
| --- | --- | --- | --- | --- | --- | --- | --- | --- | --- | --- | --- | --- | --- |
| M1 | 4 | SPB | 0.056 | 0.109 | 0.217 | 6.6 | 30.1 | 100.7 | 122.5 | 442.0 | 657.6 | -17.8 | -41.3 |
| M1 | 4 | CL | 0.036 | 0.103 | 0.148 | 33.3 | 82.8 | 99.7 | 954.4 | 730.9 | 380.6 | 8.0 | 44.1 |
| M1 | 8 | SPB | 0.050 | 0.154 | 0.215 | 25.0 | 107.0 | 142.3 | 515.0 | 795.0 | 595.0 | -14.0 | 16.8 |
| M1 | 8 | CL | 0.079 | 0.137 | 0.192 | 61.5 | 114.3 | 150.1 | 779.9 | 911.1 | 638.0 | -10.4 | 27.1 |
| M1 | 12 | SPB | 0.064 | 0.154 | 0.227 | 18.1 | 82.0 | 144.3 | 273.8 | 690.3 | 847.4 | -26.7 | -50.9 |
| M1 | 12 | CL | 0.057 | 0.164 | 0.212 | 30.0 | 125.7 | 152.4 | 527.9 | 887.2 | 632.9 | -20.3 | 21.4 |
| M1 | 16 | SPB | 0.044 | 0.085 | 0.226 | 17.0 | 56.7 | 263.6 | 409.1 | 967.1 | 1470.5 | -24.6 | -67.6 |
| M1 | 16 | CL | 0.014 | 0.041 | 0.198 | 9.0 | 37.0 | 256.1 | 688.2 | 1062.6 | 1394.9 | -5.3 | -18.7 |
| M1 | 20 | SPB | 0.069 | 0.133 | 0.236 | 20.3 | 66.8 | 176.8 | 271.6 | 516.2 | 955.8 | -16.9 | -75.6 |
| M2 | 4 | CL | 0.016 | 0.044 | 0.105 | 65.1 | 123.8 | 289.7 | 4170.1 | 2023.6 | 2710.4 | 34.0 | 3.6 |
| M2 | 4 | SPB | 0.015 | 0.040 | 0.109 | 51.0 | 97.8 | 267.8 | 3590.1 | 1843.4 | 2453.6 | 26.1 | 1.5 |
| M2 | 8 | SPB | 0.031 | 0.074 | 0.149 | 59.1 | 148.8 | 356.8 | 1736.0 | 1668.2 | 2803.1 | 2.1 | -82.2 |
| M2 | 8 | CL | 0.018 | 0.060 | 0.144 | 53.7 | 147.4 | 379.2 | 3004.8 | 2239.2 | 2774.9 | 14.1 | -18.2 |
| M2 | 12 | SPB | 0.027 | 0.100 | 0.177 | 49.3 | 210.7 | 455.8 | 1822.7 | 2198.1 | 3191.5 | -10.1 | -109.9 |
| M2 | 12 | CL | 0.018 | 0.075 | 0.157 | 54.5 | 193.5 | 421.5 | 3145.5 | 2440.9 | 2810.2 | 12.7 | -15.0 |
| M3A | 4 | CL | 0.022 | 0.160 | 0.431 | 53.7 | 53.6 | 103.1 | 2409.6 | 0.0 | 181.5 | 53.9 | 24.9 |
| M3A | 4 | SPB | 0.027 | 0.172 | 0.518 | 51.3 | 51.6 | 112.8 | 1936.9 | 2.4 | 177.7 | 51.7 | 21.5 |
| M3A | 8 | SPB | 0.091 | 0.313 | 0.535 | 65.4 | 65.4 | 78.4 | 719.4 | 0.0 | 58.6 | 65.5 | 47.2 |
| M3A | 8 | CL | 0.097 | 0.310 | 0.527 | 70.5 | 70.5 | 95.6 | 728.0 | 0.0 | 116.5 | 70.9 | 34.8 |
| M3A | 12 | CL | 0.142 | 0.350 | 0.551 | 106.3 | 106.3 | 130.1 | 748.9 | 0.0 | 116.5 | 106.6 | 65.8 |
| M3A | 12 | SPB | 0.137 | 0.343 | 0.551 | 106.2 | 106.2 | 123.1 | 787.7 | 0.0 | 83.3 | 107.6 | 79.1 |
| M3B | 4 | SPB | 0.062 | 0.120 | 0.164 | 66.0 | 161.2 | 297.6 | 958.3 | 1738.4 | 3070.2 | -48.6 | -207.9 |
| M3B | 4 | CL | 0.061 | 0.099 | 0.148 | 102.5 | 186.2 | 320.3 | 1715.6 | 2226.2 | 2769.3 | -31.1 | -84.9 |
| M3B | 8 | SPB | 0.047 | 0.103 | 0.172 | 34.1 | 107.2 | 288.0 | 888.6 | 1646.0 | 3259.4 | -35.7 | -201.8 |
| M3B | 8 | CL | 0.041 | 0.079 | 0.169 | 58.0 | 113.0 | 309.7 | 1417.5 | 1450.0 | 2173.5 | -1.3 | -58.5 |
| M3B | 12 | SPB | 0.063 | 0.144 | 0.233 | 18.2 | 91.1 | 258.8 | 307.6 | 891.5 | 1900.4 | -36.6 | -182.3 |
| M3B | 12 | CL | 0.052 | 0.125 | 0.221 | 22.1 | 87.5 | 261.6 | 430.1 | 898.5 | 1843.9 | -24.5 | -142.8 |
| M3B | 16 | SPB | 0.067 | 0.136 | 0.201 | 38.4 | 135.6 | 312.2 | 570.3 | 1409.8 | 2748.7 | -56.6 | -239.3 |
| M3B | 16 | CL | 0.064 | 0.114 | 0.180 | 80.2 | 167.6 | 361.2 | 1256.1 | 1744.5 | 2955.3 | -31.1 | -169.4 |
| M3B | 20 | SPB | 0.092 | 0.160 | 0.218 | 39.1 | 117.3 | 250.1 | 427.7 | 1139.9 | 2292.5 | -65.3 | -249.8 |
| M3B | 20 | CL | 0.058 | 0.132 | 0.207 | 46.0 | 127.1 | 267.1 | 798.6 | 1091.7 | 1854.8 | -16.9 | -117.5 |
| M4 | 4 | SPB | 0.110 | 0.175 | 0.278 | 51.8 | 121.7 | 336.8 | 462.6 | 1109.1 | 2291.0 | -71.0 | -278.3 |
| M4 | 4 | CL | 0.086 | 0.129 | 0.243 | 70.8 | 134.4 | 495.0 | 952.7 | 2221.9 | 3160.7 | -109.4 | -230.8 |
| M4 | 8 | SPB | 0.137 | 0.221 | 0.298 | 56.8 | 149.4 | 338.5 | 415.1 | 1116.3 | 2446.1 | -96.3 | -389.6 |
| M4 | 8 | CL | 0.064 | 0.141 | 0.216 | 45.9 | 158.3 | 394.4 | 712.6 | 1478.2 | 3140.1 | -49.4 | -282.9 |
| M4 | 12 | SPB | 0.164 | 0.249 | 0.354 | 46.8 | 115.3 | 293.6 | 285.2 | 812.8 | 1692.6 | -86.7 | -305.4 |
| M4 | 12 | CL | 0.102 | 0.176 | 0.280 | 51.6 | 120.3 | 308.9 | 509.3 | 931.4 | 1816.3 | -43.1 | -198.8 |
| M4 | 16 | SPB | 0.184 | 0.273 | 0.363 | 40.6 | 118.2 | 286.3 | 221.2 | 865.0 | 1877.3 | -118.2 | -394.9 |
| M4 | 16 | CL | 0.128 | 0.184 | 0.266 | 56.0 | 117.3 | 284.8 | 442.2 | 1094.9 | 2043.9 | -83.5 | -258.4 |
| M4 | 20 | SPB | 0.197 | 0.306 | 0.404 | 35.8 | 106.2 | 261.3 | 182.2 | 657.4 | 1586.8 | -93.7 | -378.1 |
| M4 | 20 | CL | 0.127 | 0.195 | 0.283 | 49.5 | 112.0 | 262.1 | 397.7 | 918.4 | 1768.2 | -66.3 | -232.4 |
